# Associations between demographic characteristics and Big Five personality traits after anterior cruciate ligament injury: A registry‐based cross‐sectional study

**DOI:** 10.1002/jeo2.70967

**Published:** 2026-11-04

**Authors:** Ramana Piussi, Jakob Lindskog, Andreas Ivarsson, Rebecca Hamrin Senorski, Jens Augustsson, Johan Högberg, Axel Sundberg, Eric Hamrin Senorski

**Affiliations:** ^1^ Sahlgrenska Sports Medicine Center Gothenburg Sweden; ^2^ Unit of Physiotherapy, Department of Health and Rehabilitation, Institute of Neuroscience and Physiology, Sahlgrenska Academy University of Gothenburg Gothenburg Sweden; ^3^ Centre of Research on Welfare Health and Sport (CVHI) Halmstad University Halmstad Sweden; ^4^ Department of Sport Science and Physical Education University of Agder Kristiansand Norway

**Keywords:** Big‐5, knee, patient‐reported outcome, personality, questionnaires

## Abstract

**Purpose:**

The purpose of this study was to investigate associations between demographic characteristics and personality traits, measured with the Big Five Inventory‐2 (BFI‐2), in patients after anterior cruciate ligament (ACL) injury. A secondary exploratory purpose was to compare demographic characteristics across groups defined by predominant personality trait.

**Methods:**

Data for this cross‐sectional study were obtained from a Swedish rehabilitation‐specific registry. Patients aged 15–65 years who completed the Swedish version of the BFI‐2 were included. The five BFI‐2 domains (extraversion, agreeableness, conscientiousness, open‐mindedness and negative emotionality) were analysed as continuous outcomes using multivariable linear regression with sex, age at injury or ACL reconstruction, body mass index (BMI), number of ACL injuries and preinjury Tegner activity level as predictors. Predominant personality trait groups were compared in a secondary exploratory analysis.

**Results:**

A total of 782 patients were included, of whom 692 had complete data for the regression analyses. Female sex was associated with higher scores in all five BFI‐2 domains (all *p* ≤ 0.045). Older age was associated with higher extraversion, conscientiousness and open‐mindedness and lower negative emotionality (all *p* ≤ 0.006). Higher BMI was associated with lower conscientiousness (*p* = 0.004) and higher open‐mindedness (*p* = 0.049). The models explained 2.7%–13.0% of the variance in personality scores. In the secondary analysis, Agreeableness was the most common predominant trait, and patients classified as predominantly open‐minded were older than patients in the other groups.

**Conclusions:**

Sex and age were associated with several BFI‐2 personality traits after ACL injury, whereas BMI showed only small associations. However, demographic characteristics explained little of the variance in personality scores, indicating that personality cannot be reliably inferred from demographic characteristics alone.

**Level of Evidence:**

Level III.

**Abbreviations**
ACL: anterior cruciate ligament
ACLR: anterior cruciate ligament reconstruction
BFI: Big Five Inventory
BFI‐2: Big Five Inventory‐2
BMI: body mass index
cm: centimeters
HT: Hamstring tendon
IKDC: International Knee Documentation Committee Subjective Knee Score
IQR: interquartile range
kg: kilograms
KOOS: Knee injury and Osteoarthritis Outcome Score
m: metres
n: number
PROs: patient‐reported outcomes
PT: patellar tendon—in the graft table
Q–Q: quantile–quantile
rec: reconstruction
RECORD: REporting of studies Conducted using Observational Routinely collected Data
SORT: strength of recommendation taxonomy
SPSS: statistical product and service solutions
Tegner: Tegner activity scale

## INTRODUCTION

An anterior cruciate ligament (ACL) injury is a traumatic sports‐related knee injury [26] with an annual incidence of 0.05 per 1000 player‐exposures [10]. Regardless of the treatment choice, that is, rehabilitation alone or ACL reconstruction and subsequent rehabilitation [9, 12], an ACL injury and its treatment can be a major challenge. For instance, persistent differences in quadriceps strength symmetry that exceed 10% have been observed 5 years after ACL reconstruction [14], and have been associated with poorer knee function and reduced return‐to‐sport rates [29]. In addition, patients with a history of ACL injury may experience reduced knee‐related quality of life up to 35 years after injury [20]. Personality traits influence emotional regulation, stress reactivity and coping strategies, thereby shaping how individuals adapt to demanding situations such as rehabilitation after injury [1, 11, 23]. Personality traits are patterns of behaviour, cognition and emotion, that tend to remain consistent across time and situations and play an important role in how patients cope with recovery demands [3].

One commonly used model to study personality traits is ‘the Big Five’, which encompasses the traits extraversion, agreeableness, conscientiousness, openness and neuroticism [33]. The model was empirically derived through lexical and factor‐analytic processes that identified the most consistent dimensions of human personality across languages and cultures [15]. The Big Five offers a valuable framework to understand how different individual personality traits may affect rehabilitation outcomes. Individuals high in Neuroticism may experience elevated fear and lower confidence, which could impede their rehabilitation progress [27, 34], whereas individuals high in Extraversion may engage more confidently and proactively in rehabilitation tasks [2].

Within the field of ACL rehabilitation, personality traits have been reported to affect adherence to rehabilitation, where agreeableness, conscientiousness and openness have been positively associated with increased adherence [17]. Furthermore, neuroticism has been negatively correlated with subjective knee scores measured with the International Knee Documentation Committee Subjective Knee Score (IKDC) and the Knee injury and Osteoarthritis Outcome Score (KOOS) at 3 and 6 months after surgery, while conscientiousness had a positive correlation with the IKDC and KOOS [35]. Individuals with a predominant personality trait of conscientiousness have been reported with higher odds of success in a balance test, where individuals predominant in neuroticism had lower odds [35]. These findings suggest that personality traits influence both behavioural engagement in rehabilitation and perceived recovery, and highlight the potential value personality assessment in rehabilitation.

Age‐ and sex‐related patterns have been well documented for Big Five personality traits in general populations [15, 30], but their expression in an injured, physically active cohort is unknown. Therefore, as an explorative step, we aimed to compare demographic characteristics across predominant personality traits in a large rehabilitation registry cohort of individuals with ACL injury. Thus, the purpose of this study was to investigate associations between demographic characteristics and personality traits, measured with the BFI‐2, in patients after ACL injury. A secondary exploratory purpose was to compare demographic characteristics across groups defined by predominant personality trait.

## MATERIALS AND METHODS

This study was reported according to the REporting of studies Conducted using Observational Routinely collected Data (RECORD) statement [4].

The present study utilised data from Project ACL, a Swedish rehabilitation‐specific registry designed to enhance care for individuals with ACL injuries, regardless of treatment approach [16]. Participation is voluntary and informed consent is acquired prior to participation in Project ACL. Patients in Project ACL are invited to undergo regular evaluations with muscle function tests and PROs at 10 weeks, 4, 8, 12, 18, 24 months and every fifth year after ACL injury or reconstruction. Upon registration in Project ACL, patients provide demographical variables such as sex, age, body weight, and height. Project ACL has ethical approval from the Regional Ethical Review Board in Gothenburg, Sweden (registration numbers: 265‐13 and T023‐17), and from the Swedish Ethical Review Authority (registration numbers: 2020‐02501, 2024‐08724‐01).

### Data collection

At each follow‐up in Project ACL, evaluation consists of answers to PROs and muscle function tests. From March 2025, new registered patients were asked to complete the Swedish version of the BFI‐2 questionnaire to assess personality traits. All patients registered before March 2025 were sent one e‐mail with the BFI‐2 questionnaire, and one reminder 3 weeks after, in case of no response. Answers to the BFI‐2 questionnaire were collected via a web‐based platform.

#### The Big Five Inventory‐2 (BFI‐2)

The BFI model encompasses the personality traits: Extraversion, agreeableness, conscientiousness, openness and neuroticism. With the development of the BFI‐2, there has been some re‐wording of the personality traits [15, 30]. In fact, the BFI‐2 is a measure of the Big Five personality traits that now read: Extraversion, agreeableness, conscientiousness, open‐mindedness and negative emotionality, as well as 15 more specific facet traits. Facet traits represent narrower subcomponents of the broader personality trait (Table 1) [15, 30].

**Table 1:** Personality traits with a definition and facet traits [15, 30].

Personality trait	Definition	Facet traits
Extraversion	Characterised by traits such as sociable, energetic, assertive and talkative; reflects the extent to which people are outgoing, expressive and experience positive emotions.	– Sociability
		– Assertiveness
		– Energy level
Agreeableness	Defined by traits such as kind, cooperative, trusting and generous, reflects interpersonal tendencies, especially in terms of compassion and concern for others.	– Compassion
		– Respectfulness
		– Trust
Conscientiousness	Includes traits such as organised, reliable, thorough, and planful, and pertains to impulse control, goal‐directed behaviour and persistence.	– Organisation
		– Productiveness
		– Responsibility
Open‐mindedness	Includes traits such as imaginative, curious, open‐minded, and artistic, reflects intellectual curiosity, creativity and preference for novelty and variety.	– Intellectual curiosity
		– Aesthetic sensitivity
		– Creative imagination
Negative emotionality	Captures traits such as anxious, moody, easily upset and reflects emotional instability and the tendency to experience negative emotions.	– Anxiety
		– Depression
		– Emotional volatility

The BFI‐2 expands on the original BFI [18] by including facet traits for each trait. Each of the five traits is divided into three facet traits, making a total of 15 facet traits. The BFI‐2 consists of 60 sentences, where patients rate on a scale between 1 (strongly disagree) to 5 (strongly agree). There are four sentences per facet trait, for a total of 12 sentences per personality trait. For each of the five personality traits, a mean score is calculated with higher scores indicating higher levels of the respective personality trait [30]. In addition to the continuous personality trait scores, patients were exploratively classified according to their highest‐scoring personality trait, referred to in the present study as the predominant personality trait.

Internal consistency for the five personality trait scales in the BFI‐2 is high, with Cronbach's alpha values between 0.83 and 0.85 [30]. The 15 facet scales have also been reported with acceptable to good internal consistency, with alpha coefficients that range from 0.66 to 0.85 [30]. Test–retest reliability has been reported with coefficients of 0.76–0.84 and facet‐level coefficients ranged 0.66–0.85 [30]. In terms of validity, the BFI‐2 has been reported with good construct validity and discriminant validity [30].

The Swedish version used in this study has comparable reliability with the original version and has good criterion validity through meaningful associations with several relevant constructs, which include psychological well‐being, attachment styles and social dominance orientation [36].

### Variables

Variables used in the present study included the five continuous BFI‐2 personality trait scores: Extraversion, agreeableness, conscientiousness, open‐mindedness and negative emotionality. For the secondary exploratory analysis, the trait with the highest mean score was presented as the predominant personality trait. The distribution of facet trait scores was also presented descriptively. Further variables used included sex, age at injury or reconstruction, number of ACL injuries sustained, body height, body weight, body mass index (BMI), graft choice for ACL reconstruction, and pre‐injury activity level, measured with the Tegner activity scale [31]. The original Tegner scale ranges from 0 to 10, however, Project ACL uses a modified version which starts from level 1, where level 0 (disability due to sick leave) was removed. Higher Tegner levels indicate higher levels of knee‐strenuous activity. The Tegner has acceptable test‐retest reliability (intraclass correlation coefficient = 0.8) [8].

### Patients included in the present study

Patients registered in Project ACL who responded to all questions in the BFI‐2 were eligible for inclusion. Patients younger than 15 years or older than 65 years were excluded.

### Study execution

Personality trait data from the BFI‐2 were primarily analysed as five continuous domain scores: Extraversion, agreeableness, conscientiousness, open‐mindedness and negative emotionality. For the secondary exploratory analysis, each patient was classified according to the personality trait with the highest mean score, defined as the predominant personality trait. Demographic characteristics were then compared across the five predominant personality trait groups.

### Outcome

The main outcome of interest was the association between demographic characteristics and the five continuous BFI‐2 personality trait scores. A secondary outcome was the exploratory comparison of demographic characteristics across the five predominant personality trait groups.

### Statistical analysis

Descriptive statistics were calculated for all variables. Variable distribution across groups was assessed with the Shapiro–Wilk test and by visual inspection of histograms and Q–Q plots. For the main outcome of this study, five separate multivariable linear regression analyses were performed, with extraversion, agreeableness, conscientiousness, open‐mindedness and negative emotionality scores as dependent variables. Sex, age at injury or reconstruction, BMI, number of ACL injuries, and pre‐injury Tegner activity level were included as independent variables in each model. Regression analyses were performed using complete cases. Regression assumptions were assessed by inspection of residual plots and Q–Q plots. Multicollinearity was assessed using variance inflation factors (VIF), and influential observations were assessed using Cook's distance. Heteroscedasticity was assessed using residual plots and the Breusch–Pagan test [7]. HC3 heteroscedasticity‐consistent standard errors were used to calculate 95% confidence intervals (CIs) and *p*‐values. Regression coefficients (*B*) with 95% CIs were reported. To account for multiple testing across the five regression models, *p*‐values were adjusted using the Benjamini–Hochberg false discovery rate procedure [5]. The coefficient of determination (*R* ^2^) was reported for each model to describe the proportion of variance in the BFI‐2 domain score explained by the included demographic variables. For the secondary exploratory analysis, differences in demographic variables between personality trait groups were analysed with the Kruskal–Wallis tests for non‐normally distributed continuous variables since variables were non‐normally distributed. For categorical variables, Chi‐square tests were used, and when expected cell counts were <5, Fisher's exact test (Monte‐Carlo simulation for larger tables) was used instead. When overall group differences were observed, post‐hoc comparisons were performed using Dunn's test with Holm–Bonferroni correction (Kruskal–Wallis), or *z*‐tests for column proportions with Bonferroni correction to control multiple comparisons (chi‐square). Analyses were conducted using the Statistical Product and Service Solutions (SPSS, IBM Corp. Released 2017. IBM SPSS Statistics for Windows, Version 27.0. Armonk, NY: IBM Corp.), and with R (R Core Team. R: A language and environment for statistical computing. R Foundation for Statistical Computing, Vienna, Austria. Version 4.2.0; 2022. Available at: https://www.R-project.org/), with a significance level set at *p* < 0.05.

## RESULTS

Of the 4715 patients invited to complete the BFI‐2, 812 completed all items, corresponding to a complete‐response rate of 17.2%. After application of the age criteria, 782 patients (men *n* = 311; 39.8%), on average 29.3 ± 11.7 years old, were included in the present study (Figure 1). Of the 782 patients, 7% (*n* = 60) were treated with rehabilitation alone, and 93% (*n* = 722) were treated with ACL reconstruction.

**Figure 1:**
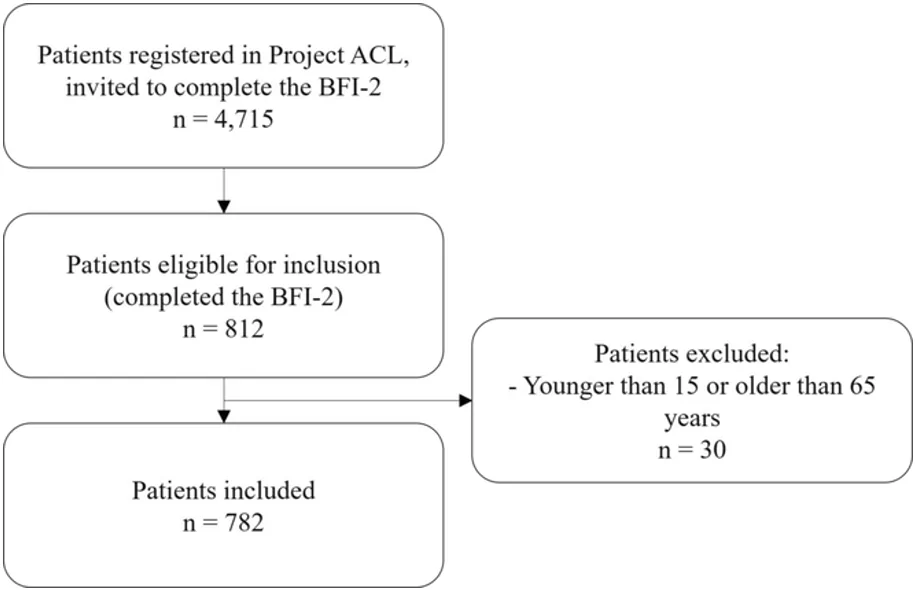
Flowchart of inclusion and exclusion. ACL, anterior cruciate ligament; BFI‐2, Big Five Inventory‐2.

Complete data for all variables included in the regression models were available for 692 patients. Results from the primary analysis are presented separately for each personality trait (Table 2).

**Table 2:** Multivariable linear regression analyses.

BFI‐2 domain	Variable assessed for association	*B* (95% CI)	Adjusted *p* value	*R* ^2^
Extraversion	Women versus men	0.118 (0.033−0.203)	0.018	0.027
	Age at injury/reconstruction, per 5 years	0.030 (0.011−0.048)	0.006	
	BMI, per 1 kg/m^2^	−0.003 (−0.020 to 0.014)	0.759	
	Number of ACL injuries, per additional injury	−0.039 (−0.149 to 0.071)	0.568	
	Pre‐injury Tegner, per level	0.014 (−0.011 to 0.038)	0.448	
Agreeableness	Women versus men	0.187 (0.121−0.254)	<0.001	0.050
	Age at injury/reconstruction, per 5 years	0.006 (−0.011 to 0.021)	0.568	
	BMI, per 1 kg/m^2^	−0.005 (−0.015 to 0.005)	0.517	
	Number of ACL injuries, per additional injury	−0.005 (−0.077 to 0.068)	0.902	
	Pre‐injury Tegner, per level	0.009 (−0.010 to 0.027)	0.529	
Conscientiousness	Women versus men	0.111 (0.032–0.189)	0.018	0.061
	Age at injury/reconstruction, per 5 years	0.050 (0.033–0.067)	<0.001	
	BMI, per 1 kg/m^2^	−0.019 (−0.031 to −0.008)	0.004	
	Number of ACL injuries, per additional injury	0.069 (−0.023 to 0.161)	0.251	
	Pre‐injury Tegner, per level	0.020 (−0.000 to 0.041)	0.108	
Open‐Mindedness	Women versus men	0.115 (0.020−0.210)	0.045	0.060
	Age at injury/reconstruction, per 5 years	0.049 (0.026−0.072)	<0.001	
	BMI, per 1 kg/m^2^	0.015 (0.002−0.029)	0.049	
	Number of ACL injuries, per additional injury	−0.042 (−0.147 to 0.064)	0.547	
	Pre‐injury Tegner, per level	−0.006 (−0.033 to 0.022)	0.753	
Negative emotionality	Women versus men	0.399 (0.304–0.495)	<0.001	0.130
	Age at injury/reconstruction, per 5 years	−0.055 (−0.078 to −0.033)	<0.001	
	BMI, per 1 kg/m^2^	0.015 (−0.001 to 0.031)	0.136	
	Number of ACL injuries, per additional injury	0.055 (−0.068 to 0.177)	0.529	
	Pre‐injury Tegner, per level	−0.011 (−0.039 to 0.017)	0.547	

*Note*: Estimates are based on HC3 robust standard errors. *p*‐Values were adjusted for multiple testing using the Benjamini–Hochberg false discovery rate procedure (*n* = 692).

Abbreviations: ACL, anterior cruciate ligament; *B*, unstandardised regression coefficient; BFI‐2, Big Five Inventory‐2; BMI, body mass index; CI, confidence interval; *R* ^2^, coefficient of determination; kg/m^2^, kilogram per square metre.

### Extraversion

Female sex and older age at injury or ACL reconstruction were associated with higher extraversion scores. Women had, on average, a 0.118‐point higher extraversion score than men (*B* = 0.118, 95% CI 0.033–0.203, *p* = 0.018), while each 5‐year increase in age was associated with a 0.030‐point higher extraversion score (*B* = 0.030, 95% CI 0.011–0.048, *p* = 0.006). The model explained 2.7% of the variance in extraversion scores (*R* ^2^ = 0.027).

### Agreeableness

Female sex was also associated with higher agreeableness scores (*B* = 0.187, 95% CI 0.121–0.254, *p* < 0.001). The model explained 5.0% of the variance in agreeableness scores (*R* ^2^ = 0.050).

### Conscientiousness

Female sex (*B* = 0.111, 95% CI 0.032–0.189, *p* = 0.018) and each 5‐year increase in age (*B* = 0.050, 95% CI 0.033–0.067, *p* < 0.001) were associated with higher scores, whereas higher BMI was associated with lower scores (*B* = −0.019 per 1 kg/m^2^, 95% CI −0.031 to −0.008, *p* = 0.004). The model explained 6.1% of the variance in Conscientiousness scores (*R* ^2^ = 0.061).

### Open‐mindedness

Higher scores were associated with female sex (*B* = 0.115, 95% CI 0.020–0.210, *p* = 0.045), older age (*B* = 0.049 per 5 years, 95% CI 0.026–0.072, *p* < 0.001) and higher BMI (*B* = 0.015 per 1 kg/m^2^, 95% CI 0.002–0.029, *p* = 0.049). The model explained 6.0% of the variance in open‐mindedness scores (*R* ^2^ = 0.060).

### Negative emotionality

Women had higher scores than men (*B* = 0.399, 95% CI 0.304–0.495, *p* < 0.001), while older age was associated with lower negative emotionality scores (*B* = −0.055 per 5 years, 95% CI −0.078 to −0.033, *p* < 0.001). The model explained 13.0% of the variance in negative emotionality scores (*R* ^2^ = 0.130).

Number of ACL injuries and pre‐injury Tegner activity level were not significantly associated with any of the five BFI‐2 domains after adjustment for multiple testing.

For the secondary exploratory analysis, out of the 782 patients, 55.4% (*n* = 433) had agreeableness as a predominant personality trait, 26.5% (*n* = 207) had conscientiousness, 10% (*n* = 78) had extraversion, 7% (*n* = 55) had open‐mindedness, and 1.1% (*n* = 9) had negative emotionality. Each predominant personality trait was supported by three facet traits. Figure 2 presents the facet traits for each predominant personality trait, for example, sociability, energy level and assertiveness are the facet traits for the extraversion personality trait. Figure 2 also presents the spread of mean ratings for each of the predominant personality traits.

**Figure 2:**
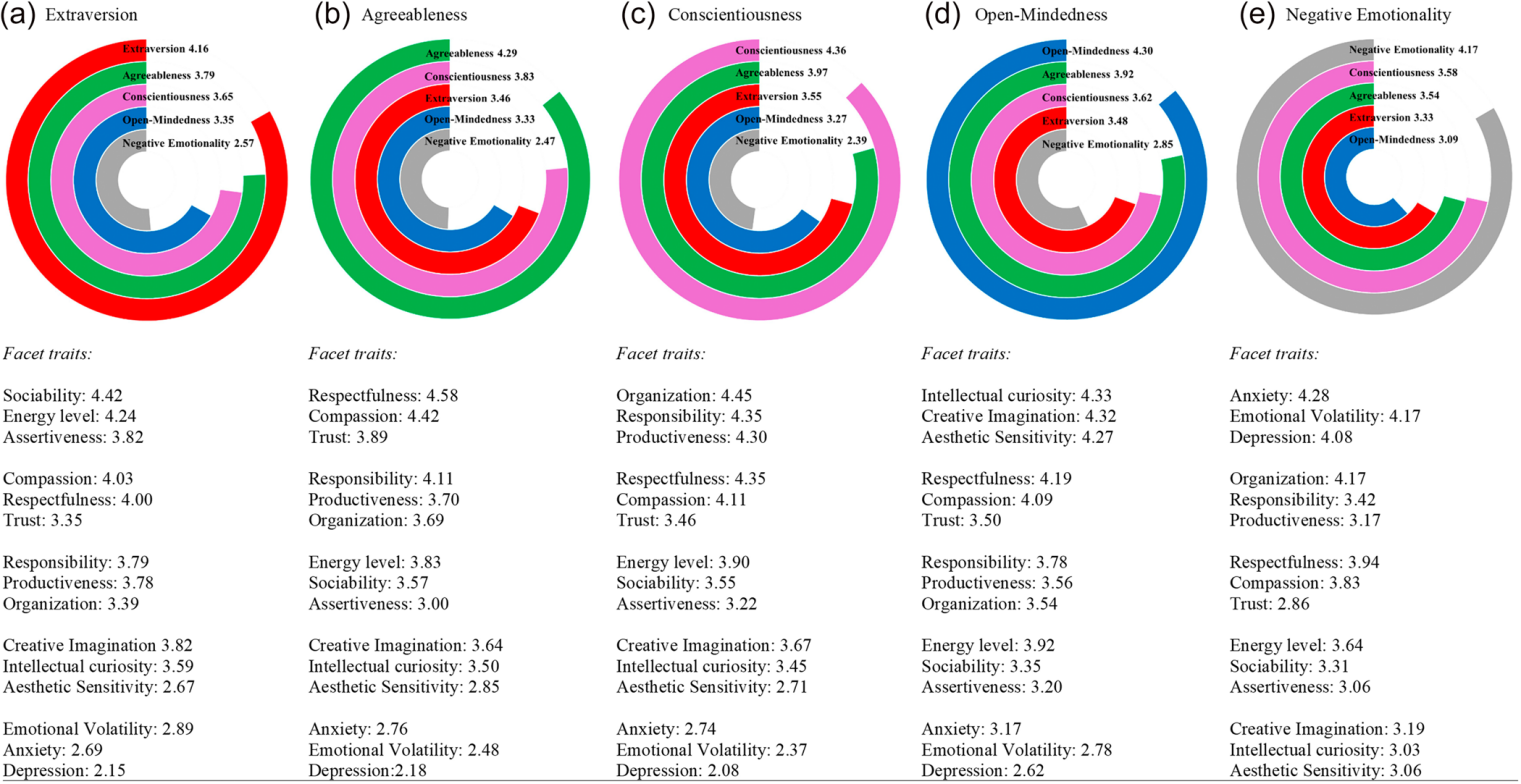
Distribution of personality trait scores in each group of patients, divided according to predominant personality trait (one per column). Each column represents one predominant personality trait. Within each column, the first three facet traits displayed correspond to the predominant personality trait for that group. The remaining twelve facet traits represent the other four predominant traits (three facets per trait) and are ordered according to descending mean predominant trait score within that group. Mean facet scores are presented descriptively.

Table 3 reports the results from the analysis on demographic differences across predominant personality traits.

**Table 3:** Comparison of demographic variables across groups.

	Extraversion (*n* = 78)	Agreeableness (*n* = 433)	Conscientiousness (*n* = 207)	Open‐mindedness (*n* = 55)	Negative emotionality (*n* = 9)	*p*‐Value
Women, *n* (%)	43 (55%)	267 (61%)	116 (56%)	36 (65%)	9 (100%)	0.054
Age at injury/ACL rec (years)	27.7 (21.3)^b^	23.5 (15.6)^b^, ^c^	27.3 (21.3)^b^, ^c^	33.2 (14.2)^b^	21.9 (8.9)^b^	<0.001^a^
Height (cm)	173.5 (17.5)	173 (14)	173 (13)	170 (11.5)	169 (9)	0.242
Weight (kg)	74.5 (19)	73 (17)	71 (16)	72 (16)	62 (7)	0.102
BMI (kg/m^2^)	23.6 (4.4)	23.8 (2.9)	23.5 (3.7)	24.4 (3.4)	22.3 (2.1)	0.033^e^
Number of ACL injuries, *n* (%)						
1 injury	71 (91.1%)	372 (85.9%)	170 (82.1%)	49 (89%)	8 (88%)	0.549
2injuries	5 (6.4%)	57 (13.1%)	31 (15%)	6 (11%)	1 (12%)	
3 injuries	2 (2.5%)	4 (1%)	6 (2.9%)	0	0	
Graft for index ACLR, *n* (%)						
HT	50 (64.1%)	253 (58.4%)	123 (59.4%)	32 (58.2%)	6 (66.7%)	0.986
PT	16 (20.5%)	71 (16.4%)	32 (15.5%)	7 (12.7%)	1 (11.1%)	
Other	4 (5.1%)	12 (2.7%)	5 (2.4%)	0	0	
Missing	10 (12.8%)	67 (15.5%)	32 (15.5%)	7 (12.7%)	2 (22.2%)	
Not applicable	6 (7.7%)	30 (6.9%)	15 (7.2%)	9 (16.4%)	0	
Pre‐injury Tegner level, *n* (%)						
Median	8	8	8	6	7.5	0.056^d^
1	1 (1.3%)	3 (0.7%)	1 (0.5%)	0	1 (11.1%)	
2	1 (1.3%)	9 (2.1%)	2 (1%)	1 (1.8%)	1 (11.1%)	
3	2 (2.6%)	17 (3.9%)	7 (3.4%)	3 (5.5%)	0	
4	2 (2.6%)	21 (4.8%)	11 (5.3%)	6 (10.9%)	0	
5	5 (6.4%)	23 (5.3%)	9 (4.3%)	4 (7.3%)	0	
6	12 (15.4%)	37 (8.5%)	32 (15.5%)	12 (21.8%)	0	
7	11 (14.1%)	67 (15.5%)	32 (15.5%)	6 (10.9%)	2 (22.2%)	
8	17 (21.8%)	65 (15%)	33 (15.9%)	7 (12.7%)	1 (11.1%)	
9	12 (15.4%)	95 (21.9%)	45 (21.7%)	4 (7.3%)	0	
10	7 (9%)	41 (9.5%)	17 (8.2%)	4 (7.3%)	3 (33.3%)	
Missing	8 (10.3%)	55 (12.7%)	18 (8.7%)	8 (14.5%)	1 (11.1%)	

*Note*: Medians and IQRs reported if not otherwise stated.

Abbreviations: ACL, anterior cruciate ligament; ACLR, anterior cruciate ligament reconstruction; BMI, body mass index; cm, centimeters; HT, hamstrings tendon; IQR, interquartile range; kg, kilograms; m, metres; *n*, number; PT, patellar tendon; rec, reconstruction; Tegner, Tegner activity scale.

^a^ Post hoc comparison with Dunn's test with Holm–Bonferroni correction.

^b^ Statistical difference after Dunn's test with Holm–Bonferroni correction between open‐mindedness group and all other groups (vs. extraversion *p* = 0.0051; vs. agreeableness *p* < 0.001; vs. consenctiousness *p* = 0.0047; vs. negative emotionality *p* = 0.0132).

^c^ Statistical difference after Dunn's test with Holm–Bonferroni correction between agreeableness and conscientiousness *p* = 0.0132.

^d^ Fisher's exact test (Monte–Carlo simulation for larger tables) was used due to cell counts <5.

^e^ After Holm–Bonferroni adjustment, significant difference in BMI across groups was no longer significant.

There was a significant difference across personality trait groups in age, where patients in the open‐mindedness group were significantly older compared to patients in all other groups. There was a significant difference in BMI across groups that was no longer present after Bonferroni adjustment (Table 3).

## DISCUSSION

The main findings of this study were that sex and age at injury or ACL reconstruction were associated with several BFI‐2 personality traits. Female sex was associated with higher extraversion, agreeableness, conscientiousness, open‐mindedness and negative emotionality scores, while older age was associated with higher extraversion, conscientiousness, and open‐mindedness scores and lower negative emotionality scores. Higher BMI was associated with lower conscientiousness and higher open‐mindedness scores. However, the demographic variables explained only a small proportion of the variance in personality trait scores, ranging from 2.7% for Extraversion to 13.0% for negative emotionality. In the secondary exploratory analysis, patients classified as predominantly open‐minded were older than patients in the other predominant personality trait groups.

In this study, female sex was associated with higher scores across all five BFI‐2 domains, with the largest difference observed for negative emotionality, followed by agreeableness, which is broadly consistent with previous population‐based research [24, 28]. Women generally report higher neuroticism/negative emotionality and agreeableness than men, with smaller and less consistent differences in extraversion, conscientiousness and openness/open‐mindedness [24]. Further, Schmitt et al. [28] reported higher neuroticism, extraversion, agreeableness and conscientiousness among women across most of 55 cultures. Accordingly, the sex‐related differences observed in the present ACL cohort appear broadly consistent with established population‐level personality distributions, despite the cohort representing a clinically and physically active subgroup of the general population.

As for age, previous population‐based studies have consistently reported age‐related differences in Big Five personality traits, although the direction and magnitude of the associations vary across traits and stages of life [13, 25]. Higher conscientiousness and lower neuroticism/negative emotionality with increasing age have been reported across several large cross‐sectional and longitudinal studies [22, 32]. In contrast, findings for extraversion and openness/open‐mindedness are less consistent, and studies that include individuals with a broad adult age range have generally reported lower extraversion and openness with older age [13, 22]. The relatively young age distribution of the present cohort may therefore partly explain the positive associations observed between extraversion and open‐mindedness and age. However, age in the present study represents age at ACL injury or reconstruction rather than age at BFI‐2 assessment, and the findings should therefore not be interpreted as evidence of age‐related personality development. Nevertheless, personality traits show substantial rank‐order stability, particularly from young adulthood onward, with meta‐analytic evidence that reports that stability in personality traits increases through early life and reaches a plateau around age 25 [6]. Therefore, although the exact timing of BFI‐2 assessment relative to ACL injury or reconstruction was not available in the registry, variation in assessment timing may be less problematic for personality traits than for more time‐sensitive rehabilitation‐specific psychological constructs, such as knee‐related self‐efficacy.

Finally, higher BMI was associated with lower conscientiousness and higher open‐mindedness scores, although both associations were small. The inverse association between BMI and conscientiousness is consistent with previous research, where a meta‐analysis of nine cohort studies reported that higher conscientiousness was associated with a lower risk of obesity [19]. Given the small magnitude of the associations observed in the present study, the practical relevance of BMI in relation to personality traits appears limited.

Despite several statistically significant associations, the demographic variables explained only 2.7%–13.0% of the variance in the individual personality trait scores. Thus, although sex and age were associated with several personality traits at the group level, demographic characteristics provide limited information about an individual patient's personality profile. Clinically, age, sex, or other demographic characteristics should therefore not be used as proxies for personality. If future studies demonstrate that personality traits contribute to rehabilitation outcomes, direct assessment of personality would be required to identify potentially relevant individual differences.

In the secondary exploratory analysis, agreeableness was the most common predominant personality trait, whereas negative emotionality was rarely the highest‐scoring trait. Importantly, classification according to the highest‐scoring domain reflects the relative distribution of personality traits within an individual and does not imply that patients classified into other groups had low levels of negative emotionality. The facet‐level scores were examined descriptively and showed relatively high scores for agreeableness facets across several predominant personality trait groups, whereas open‐mindedness facets generally ranked lower (Figure 2). As no inferential comparisons were performed at the facet level, these patterns should be considered descriptive and hypothesis‐generating.

Future studies should investigate whether continuous personality trait scores are associated with clinically relevant outcomes after ACL injury, such as rehabilitation adherence, psychological responses, return to sport, or subsequent injury. Personality traits have previously been associated with behavioural and performance‐related characteristics in sport [21]. However, whether personality assessment provides clinically relevant information beyond demographic and established psychological factors remains to be determined.

## LIMITATIONS

This study has several limitations. First, the cross‐sectional design prevents causal inference, and the single BFI‐2 assessment does not allow evaluation of within‐person changes in personality over time. Second, the small size of the predominant negative emotionality group (*n* = 9) limits interpretation of the secondary exploratory analyses involving this group. Third, the exact timing of BFI‐2 assessment relative to ACL injury or reconstruction was not available in the registry and may have varied between patients. Additionally, complete‐response rate to the BFI‐2 was 17.2%, and patients who completed the questionnaire may differ systematically from non‐responders, potentially introducing selection bias and limiting generalisability. Furthermore, classification according to the predominant personality trait reduces five continuous BFI‐2 scores to a single categorical classification and therefore entails loss of information. For this reason, the categorical analyses were considered secondary and exploratory. The findings may not generalise to populations with low activity levels due to the very few patients included at low levels of the Tegner. Taken together, our results should be interpreted cautiously.

## CONCLUSION

Female sex was associated with higher extraversion, agreeableness, conscientiousness, open‐mindedness, and negative emotionality, while older age was associated with higher extraversion, conscientiousness, and open‐mindedness and lower negative emotionality. Higher BMI was associated with lower conscientiousness and higher open‐mindedness. However, demographic characteristics explained only a small proportion of the variance in personality traits and should therefore not be used to infer an individual patient's personality profile.

## PERSPECTIVE

To our knowledge, this is the first large‐scale registry study to examine Big Five personality traits in patients after ACL injury. This study shows that sex and age are associated with several Big Five personality traits after ACL injury, while BMI shows only small associations. However, demographic characteristics explained little of the variance in personality scores, indicating that personality cannot be reliably inferred from routine demographic characteristics. The findings should therefore be considered hypothesis‐generating rather than clinically actionable. Future studies should determine whether continuous personality trait scores are associated with rehabilitation adherence, psychological responses, return to sport, or subsequent injury, and whether personality provides clinically relevant information beyond established demographic and psychological factors.

## AUTHOR CONTRIBUTIONS

**Ramana Piussi**: Conceptualisation; formal analysis; funding acquisition; investigation; methodology; project administration; visualisation; writing—original draft; writing—review and editing. **Jakob Lindskog**: Data curation; visualisation; writing—review and editing. **Andreas Ivarsson**: Methodology; writing—review and editing. **Rebecca Hamrin Senorski**: Writing—review and editing. **Jens Augustsson**: Software; writing—review and editing. **Johan Högberg**: Writing—review and editing. **Axel Sundberg**: Writing—review and editing. **Eric Hamrin Senorski**: Conceptualisation; data curation; funding acquisition; investigation; methodology; project administration; resources; supervision; validation; writing—original draft; writing—review and editing.

## FUNDING INFORMATION

This work was supported by The Swedish Research Council for Sport Science, postdoctoral grant number D2025‐0183.

## CONFLICT OF INTEREST STATEMENT

The authors declare no conflicts of interest.

## ETHICS STATEMENT

Ethical approval was obtained from the Regional Ethical Review Board in Gothenburg, Sweden (registration numbers: 265‐13 and 265‐13, T023‐17), and from the Swedish Ethical Review Authority (registration numbers: 2020‐02501, 2024‐08724‐01).

## Data Availability

The data used for this manuscript are available from the corresponding author upon reasonable request.
